# Age and Sex Differences in Load‐Induced Tibial Cortical Bone Surface Strain Maps

**DOI:** 10.1002/jbm4.10467

**Published:** 2021-02-16

**Authors:** Alessandra Carriero, Behzad Javaheri, Neda Bassir Kazeruni, Andrew A Pitsillides, Sandra J Shefelbine

**Affiliations:** ^1^ Department of Biomedical Engineering The City College of New York New York NY USA; ^2^ School of Mathematics, Computer Science and Engineering, City University of London London UK; ^3^ Department of Biomedical Engineering Imperial College London London UK; ^4^ Department of Comparative Biomedical Sciences Royal Veterinary College London UK; ^5^ Department of Mechanical and Industrial Engineering and Department of Bioengineering Northeastern University Boston MA USA

**Keywords:** AGING, BIOMECHANICS, BONE, MOUSE, SEXUAL DIMORPHISM, STRAIN

## Abstract

Bone adapts its architecture to the applied load; however, it is still unclear how bone mechano‐adaptation is coordinated and why potential for adaptation adjusts during the life course. Previous animal models have suggested strain as the mechanical stimulus for bone adaptation, but yet it is unknown how mouse cortical bone load‐related strains vary with age and sex. In this study, full‐field strain maps (at 1 N increments up to 12 N) on the bone surface were measured in young, adult, and old (aged 10, 22 weeks, and 20 months, respectively), male and female C57BL/6J mice with load applied using a noninvasive murine tibial model. Strain maps indicate a nonuniform strain field across the tibial surface, with axial compressive loads resulting in tension on the medial side of the tibia because of its curved shape. The load‐induced surface strain patterns and magnitudes show sexually dimorphic changes with aging. A comparison of the average and peak tensile strains indicates that the magnitude of strain at a given load generally increases during maturation, with tibias in female mice having higher strains than in males. The data further reveal that postmaturation aging is linked to sexually dimorphic changes in average and maximum strains. The strain maps reported here allow for loading male and female C57BL/6J mouse legs in vivo at the observed ages to create similar increases in bone surface average or peak strain to more accurately explore bone mechano‐adaptation differences with age and sex. © 2021 The Authors. *JBMR Plus* published by Wiley Periodicals LLC. on behalf of American Society for Bone and Mineral Research.

## Introduction

Bone dynamically adapts its architecture in response to the applied loads. This mechano‐adaptation process is central in maintaining bone mass and ensuring sufficient bone strength. Previous clinical studies, in vitro cell culture experiments, and in vivo animal experiments have sought to explore bone's mechano‐adaptive responses.^(^ ^1^, ^2^, ^3^, ^4^, ^5^, ^6^, ^7^, ^8^, ^9^, ^10^, ^11^, ^12^, ^13^, ^14^ ^)^ The numerous animal models developed to study mechano‐adaptation have shown that the parameters of the loading regime (i.e., magnitude, frequency, rate and rest insertion) dramatically influence the ensuing bone (re)modeling.^(^ ^4^, ^5^, ^6^, ^7^, ^8^, ^15^ ^)^ These studies have found that cyclic compressive loads of 8.7–13 N applied to the tibia of the flexed hind limb in anesthetized mice elicit an osteogenic/antiresorptive response in a spatially restricted manner that correlates with the load magnitude.^(^ ^15^, ^16^ ^)^ It is assumed that such adaptation is coordinated in response to a strain‐related stimulus, reliant on magnitude,^(^ ^17^ ^)^ rate,^(^ ^18^, ^19^ ^)^ gradient,^(^ ^20^, ^21^ ^)^ fluid velocity caused by strain,^(^ ^22^, ^23^, ^24^ ^)^ or microdamage (from excessive strain).^(^ ^25^ ^)^

To measure the bone's response to applied loading, most animal models assume that adaptation is engendered in response to a peak strain stimulus.^(^ ^5^, ^6^, ^8^, ^16^, ^17^, ^18^, ^19^, ^20^, ^21^, ^25^ ^)^ Therefore, calibration is required to ensure that the applied strains are matched during in vivo loading across groups of animals that may have different bone structure and material properties. Thus, most studies have related mechano‐adaptation of bone to the strains measured experimentally using strain gauges, attached to a single location on the exposed cortical bone surface either in vivo, to measure physiologically induced strains, or in representative ex vivo loading, for calibration. Other studies have used finite element (FE) analysis to estimate bone strains, but these analyses require assumptions to be made regarding the geometry, material properties, and loading conditions. It is evident that both methods are limited in providing measurements of the load‐related strain engendered across an entire bone surface.

Recently, digital image correlation (DIC) has been used to measure high‐resolution, full‐field strains on cortical bone surfaces in the murine tibia–loading model.^(^ ^15^, ^24^, ^26^, ^27^, ^28^ ^)^ Although applied ex vivo, DIC offers a significant advance over the typical characterization of the bone's mechanical environment. DIC measures strains in inhomogeneous, anisotropic, and nonlinear materials with a complex morphology, like bone. Using ex vivo DIC in combination with an in vivo mouse tibial loading model, we have previously shown that strains engendered on the tibia surface are not homogenous but have focal regions of high strain that become more homogenous after adaptation.^(^ ^26^, ^27^ ^)^ We have also used DIC to calibrate load magnitudes to engender a strain‐matched stimulus to tibia in mice with vastly divergent ages.^(^ ^15^ ^)^ Thus, the location selected for strain calibration is extremely important in experiments exploring how bone responds to loading.

The relationship between the mechanical strains on cortical bone and its adaptation has been explored, particularly using the murine tibial–loading model, by comparing regions of bone formation from in vivo μCT, histology, and recently three‐dimensional fluorochrome mapping coupled with FE models of mechanical stimulus.^(^ ^24^, ^29^, ^30^, ^31^, ^32^, ^33^, ^34^, ^35^ ^)^ Although comparison of bone adaptation across these studies is challenging because they do not all report changes in the same morphological bone region, they have found that cortical bone in the young murine tibia is very responsive to loading, whereas controversial results have been found in aged bone. Some studies, which have used strain gauges to calibrate the applied load, found that aged bone does not exhibit efficient adaptation at similar strain magnitudes as young bone, interpreting this result as a failure of aged bone to adapt.^(^ ^36^, ^37^, ^38^ ^)^ These data have led to an assertion that bone mechano‐adaptive response is progressively and irreversibly diminished during aging. However, other studies found that the reduced adaptation of aged mice can be reversed by disuse,^(^ ^39^, ^40^, ^41^ ^)^ and recently we found that only two bouts of DIC‐calibrated peak strain‐matched (~0.57%) loading are sufficient to initiate bone adaptation in old female mouse tibia.^(^ ^15^ ^)^ This is consistent with the notion that high strains have been considered one of the most effective drivers for bone mechano‐adaptation, as observed in previous human^(^ ^42^, ^43^ ^)^ and animal studies.^(^ ^17^, ^20^, ^44^, ^45^, ^46^ ^)^ Thus, a better understanding of the mechanical stimulus that is needed to restore bone mass in both aged male and female bone, may arise from a comprehensive understanding of the mechanical environment of the bone. In this regard, information on the strain distribution across a bone surface is a key component for a correct bone loading regime at each age and for each sex.

In this study, we compare the full‐field strains map of the cortical bone surface in young, adult, and old mice of both sexes in a mouse tibial loading model using DIC. Understanding the difference in the strain map on an entire bone surface under the same loading conditions is important to relate local mechanical stimuli to local biological response in bone tissue during aging and with sex.

## Materials and Methods

Six groups of C57BL/6J mice (*N* = 5/group; Charles River Company), males and females aged 10 weeks old, 22 weeks old, and 20 months old were considered for this study. Mice were sacrificed by cervical dislocation, and left and right tibias were exposed by removing the surrounding muscles. Bones were subsequently covered with a thin layer of matt, water‐based white paint (Dupli‐Color Aqua Lackspray; Motip Dupli GmbH), and speckled with matt, acrylic black ink (Daler Rowney) using a high‐precision airbrush (SprayCraft SP50K; Shesto). Legs were loaded up to 12 N (Instron 5800; High Wycombe) at 8 N/min load rate, with a preload of 0.2 N, using custom‐built loading cups, which apply an axial load across the knee and ankle joints. During the continuous loading, images of the medial side of the speckled tibia surface were recorded at 1 N intervals using two adjustable charge‐coupled device (CCD) cameras (100‐mm lenses with 60‐mm distance rings; GOM GmbH) at a distance of 148 cm to each other and oriented to face the bone surface to provide a field of view of 15 × 12 mm with a resolution of 7.5 × 10.9 μm and a depth of focus of 1.2 mm. The sample was lit by two light‐emitting diode lamps with polarized filters. Calibration was conducted by using a high‐precision 15 mm × 12 mm panel (GOM GmbH). Postprocessing of the images was performed using DIC ARAMIS 5M  System from GOM GmbH with 19 × 19‐pixel square facets, with 15 pixels step facet, resulting in approximately 2500 measurement points on the surface of each tibia. Three images were taken in the undeformed state to allow for the determination of the amount of error (noise) during the experiments, and the surface of each tibia was imaged at least two times to show repeatable strain fields. The analysis of these ex vivo bone images determined linear strain magnitudes and distribution patterns on about 57% of the C57BL6/J mouse tibia surface (centered at 35% of the bone length—corresponding to the visible surface on the tibia bone not hidden by the cups) during aging and with sex. Peak and average strain on the medial surface of the considered region of interest (ROI) of the tibia was calculated at each 1 N load and averaged across the specimens in each group. The average strains were calculated automatically by the software; peak strains were assessed within the ROI without considering the edges that often report noise in the DIC images.

Normal distribution and homogeneity of variance of the maximum and average strain at 12 N were analyzed by the Shapiro–Wilk test and Levene test, respectively (SPSS). Differences in maximum and average strain at the different ages and for the sexes were compared using analysis of variance factorial ANOVA (because of the normalized variables) and post hoc procedures for multiple comparisons. All tests were two tailed and *p* values smaller than 0.05 were considered to be significant.

## Results

**Fig 1:**
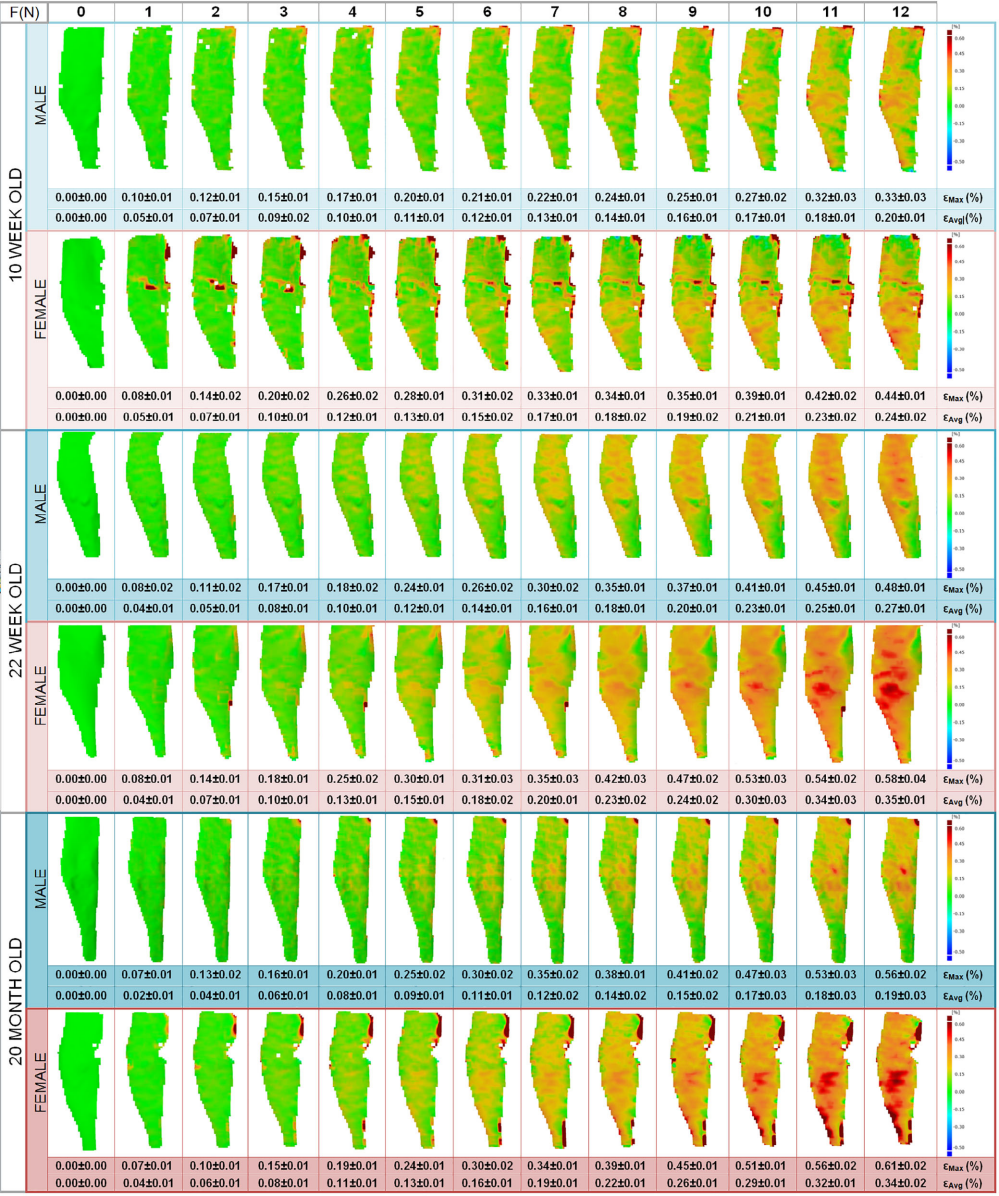
Bone surface strains for the medical surface of tibia assessed with a digital image–correlation system at 1N interval up to 12N compressive load in male and female C57BL6 mice at 10 weeks, 22 weeks, and 20 months of age. Mean and SD of peak and average strain surface is reported for each group at each load.

The spatial strain distribution over the medial side of the considered ROI of the tibia of C57BL/6J mice was measured by the DIC system based on the three components of displacement as previously described.^(^ ^26^, ^27^ ^)^ Figure 1 shows the median strain maps for male and female mice, for each group, at each 1 N of loading up to 12 N, in only the axial (loading) direction across the tibial periosteal surfaces, as transverse and shear strains were negligible in comparison. Strain maps indicate a nonuniform strain field across the surface of the tibia, with the axial compressive loads resulting in tension on the medial side of the tibia because of its curved shape, in agreement with our previous studies.^(^ ^24^, ^26^, ^27^ ^)^

**Fig 2:**
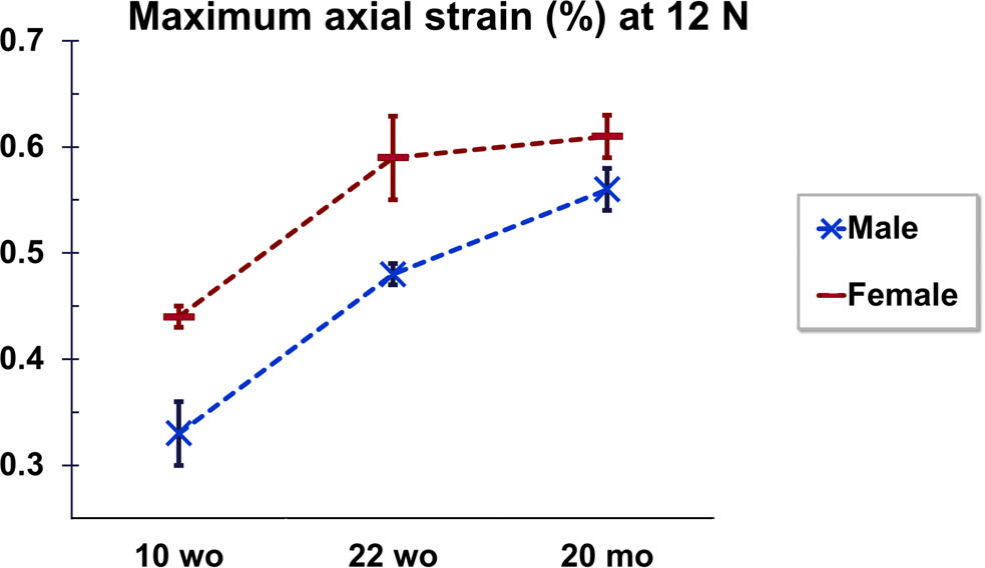
Maximum axial strain (± SD) on the tibial bone surface at 12N load shows that tensile strains dramatically increase with age (*p* < 0.001), and bone surface strains in female mice are always higher than in male mice (*p* < 0.001). Post hoc Bonferroni test reports that maximum strain values at 10 weeks old are statistically different from those at 22 weeks old and 20 months old, both in male and female mice (*p* < 0.001). Also, maximum strain values at 22 weeks of age are statistically different from those at 20 months of age, both in male and female mice (*p* < 0.05). wo, weeks old; mo, months old.

Figure 2 shows the maximum axial tibial strain at 12 N of load in male and female mice as they age. Age and sex both have a statistical significant effect on the maximum strain at 12 N (*p* < 0.001). At 10 weeks of age, tibias from male and female mice have similar strain patterns, but statistically significant different magnitudes at 12 N (peak strain of 0.33% ± 0.03% in male and 0.44% ± 0.01% in female). As the mouse ages, the strain at 12 N increases in magnitude and patterns diverge in male and female bones (Fig. 2). In particular, the medial surface of the tibia at 22 weeks of age is 0.48% ± 0.01% and 0.59% ± 0.05%, respectively, in male and female mice; in 20‐month‐old mice, the peak strain further elevates, reaching 0.56% ± 0.02% and 0.61% ± 0.02% in males and females, respectively (Fig. 2). This shows that to match the peak load‐induced strain on cortical bone to levels engendered by application of 12 N in 10‐week‐old female mouse bone (0.44%), 22‐week‐old male and female bones would need to be loaded at 11 N and 9 N, respectively, whereas 20‐month‐old male and female mouse bones would need to be loaded at 9.5 N and 9 N, respectively (Fig. 1). The DIC SD of the error (noise) was consistent throughout all the tests, at approximately 0.03%. The load‐deformation curve was similar during each repeated loading episode and for all the mice within the same group, indicating there was no failure during the load. Similar results were found for the average tibial strain at 12 N, with values always higher in female than in male mice at each age (*p* < 0.001). However, a different behavior was observed between male and females with age: though the averaged strain increased from 10 to 22 weeks old in both male and female mice, the average strain stabilized in females and decreased in males at 20 months old (Fig.1).

## Discussion

This work reveals spatial strain distribution on the medial cortical bone surface in C57BL/6J tibia during loading, allowing for a nonbiased full‐field study of the mouse bone surface mechanical environment with sex and aging. Our data show that the load‐induced surface strain magnitudes and patterns vary in C57BL/6J mice with age and sex. Axial strains on the C57BL/6J tibia surface are significantly higher with age, with tibias in female C57BL/6J mice showing larger strains than in males at high loading magnitudes (Figs. 1 and 2). This indicates that changes in cortical bone morphology and/or material properties occur in the C57BL/6J tibia during aging, which differ between sexes. Adaptation of bone to external load is central in maintaining bone mass and ensuring sufficient bone strength.^(^ ^1^, ^2^, ^3^, ^16^, ^39^, ^47^ ^)^ Using the data reported here, future studies can examine the mechano‐response of bone in aging and with sex in C57BL6/J mice with accurate strain‐match calibration.

Knowledge of age‐ and sex‐related changes in bone strain in rodents is fundamental for interpreting bone mechano‐adaptation and therapeutic and genetic interventions. Our data indicate that for a given load, surface strain increases with age in both male and female mouse tibias, and female mice always have higher strain magnitudes than males. The increase in the bone strains as mice age can be explained by changes to both the structure (geometry) and material properties that occur with aging. Skeletal maturity in C57BL6/J mice occurs at around 20 weeks of age simultaneously with an increase in bone rigidity, cross‐sectional area, and cortical thickness.^(^ ^48^, ^49^ ^)^ However, with aging, cortical bone is thinner, with similar cross‐sectional area, higher moment of area, and increased porosity.^(^ ^49^, ^50^, ^51^ ^)^ These structural variations, together with inferior bone material properties, such as the reduced flexural modulus and collagen strain,^(^ ^49^, ^51^, ^52^ ^)^ change the mechanical properties of the aging mouse bone at 20 months, increasing the strains on the bone surface. Sexually dimorphic bone structure and material properties may also explain the differences we observed in the strain magnitude and distribution on the tibia surface, with female mouse bone always having higher strains than tibias in male mice. Future work will analyze the changes in compositional, structural, and mechanical properties that underlie the age‐ and sex‐related strain variation.

Analogous changes during aging and sexual dimorphism during growth and maturity are observed in human bones,^(^ ^50^, ^53^, ^54^ ^)^ with sex‐related geometrical variation already present at 6 years of age, likely caused by differential adaptation to the mechanical loading.^(^ ^55^ ^)^ During human growth and senescence, hormone differences,^(^ ^54^, ^56^, ^57^, ^58^ ^)^ disparities in bone adaptation (moment of area) to the dissimilar mechanical environment,^(^ ^54^, ^56^, ^57^, ^59^ ^)^ and different intracortical porosity microarchitecture^(^ ^60^, ^61^, ^62^, ^63^, ^64^, ^65^, ^66^, ^67^, ^68^, ^69^ ^)^ contribute to the increased bone fragility experienced in women. Therefore, it is crucial to consider these age‐ and sex‐related differences when planning an exercise or loading regime aimed to restore bone mass in the elderly. In this regard, application of the results of this study as calibration for future studies has potential to increase our knowledge of age‐ and sex‐related differences in bone mechano‐adaptation. This may also allow for improved understanding of bone fragility as it might relate to the different bone mechano‐adaptive responses with age and sex.

The DIC data presented here were recorded at 1 N increments up to 12 N. This information can be used to load mouse bones in vivo and accurately match strains to better understand bone mechano‐adaptation and eventually define strain's localized effect on bone architecture. In a previous study, we have used DIC strain maps to calibrate in vivo loading of aged mice tibia (20 months old; 11 N) with peak strain‐matched to mature bone (22 weeks old; 12 N) and were able to initiate bone response in aged bone.^(^ ^15^ ^)^ Thus, if load is correctly strain‐matched, it is possible to stimulate a cortical mechano‐adaptive response also during aging. For years, loads applied in vivo in animal studies were strain‐matched using a single measure provided by strain gauges, and the lack in response to mechanical loading of aged bones was considered to be caused by either a failure of the osteocytes to sense load or a failure of osteoblasts to lay down new bone. With the use of the DIC strain map calibration, we were able to match the peak‐strain in mature and old bone and show that aged bone is responsive if sufficient load stimulus is applied as a trigger.^(^ ^15^ ^)^ This further shows the importance of having a full‐field strain map compared with single‐point measures obtained with strain gauges. When comparing the strain map results from our DIC with those obtained using strain gauges, it is clear that the latter significantly underestimate peak strains. Furthermore, inconsistency has been found in the results emerging from previous strain gauge studies, where a peak strain of 0.2%–0.22% was estimated in mature mice either at 8 N (20 weeks old^(^ ^70^ ^)^) or at 11.5–12 N (26 weeks old,^(^ ^71^ ^)^ 20 weeks old,^(^ ^34^ ^)^ 16 weeks old^(^ ^36^ ^)^). Our results show an average strain of 0.2% at 8 N for female adult bone (22 weeks old), whereas at 12 N the average strain is significantly higher (0.35%) than those estimated by strain gauges.^(^ ^34^, ^36^, ^71^ ^)^ Strain measurement is highly sensitive to measurement location, particularly in irregular, heterogeneous, and anisotropic material like bone. Strain gauge measurements are likely not taken at the exact peak strain site, and gauge location on bone significantly affects strain measurement.^(^ ^26^ ^)^ The results presented in this study are in agreement with previous full–field bone surface strain measurements taken during tibial loading in C57BL6/J mice.^(^ ^24^, ^26^, ^27^ ^)^ Our studies and others show divergent strains in mouse breeds known to have different material properties at the same age.^(^ ^28^, ^72^, ^73^, ^74^, ^75^ ^)^

Bone is often considered a linear material, in which strain at each single location exhibits a linear relationship with the applied loads. We have shown this is true at discrete points on the surface of the bone.^(^ ^26^ ^)^ In this study, the relationships between load and average strain on the bone surface of interest are nonlinear. Tibial curvature and irregular geometry, as well as heterogeneity in local mineralization (and stiffness) levels, determine the linear load‐strain relationship at each bone location, for which the slopes may indeed differ. With small loads, the strains on the bone surface are small and thus differences may be less evident, but with increased loads certain bone locations may experience much more strain than others. Thus the relationship between applied load and average strain on the surface of interest will not necessarily be linear. This is an important aspect of the heterogeneity in bone surface strains revealed by DIC that would not be appreciable using strain gauges.

Our studies have shown that when aged mice are loaded to match peak strain, adaptation occurs; when they are loaded to match average strain, no adaptation occurs.^(^ ^15^ ^)^ The data presented here and elsewhere^(^ ^15^ ^)^ show the importance of evaluating the loads to apply on bone prior to an in vivo load‐adaptation study. They also highlight the likely necessity, in longitudinal studies, for animals to be euthanized at different ages to allow for such calibration. Herein, we generated cortical bone strain maps for tibias of C56BL6/J mice at three ages in both sexes, and five mice per group were sufficient to reveal statistical significant differences. Our previous studies on aging and knock‐out mice^(^ ^15^, ^24^, ^27^, ^28^, ^75^ ^)^ detected tibia bone strain differences in groups containing only four bones. However, it is not possible to draw conclusions from these studies on the actual number of mice that will be needed for each bone type and mouse strain examination at different ages. Bone geometry, internal structure, and material properties will always be specimen‐specific, and differences between specimens may become larger with age, even for inbred animals kept under identical conditions. Therefore, future studies using different mouse strains and/or any other condition (animal, treatment, age group, sex, etc.) will need to include an identical group just to provide accurate strain values for their specific calibration, and to determine the sample number required for this bone strain assessment.

The presented DIC maps can further be used to validate FE models of the tibia loading in C57BL6/J mice, for example, examining the mechanical stimulus for bone adaptation.^(^ ^24^, ^35^ ^)^ Using DIC strain maps, we validated a FE model of the bone and correlated the mechanical stimuli to the ensuing spatial changes in osteogenic activity.^(^ ^24^ ^)^ We found that fluid velocity predicts bone adaptation in adult bone both periosteally and endosteally, whereas strain energy density fails to predict endosteal bone formation.^(^ ^24^ ^)^ Future studies will further investigate the mechanical stimulus in bone adaptation with age and sex to guide the development of loading protocols to direct bone formation to a specific site in osteopenia.

## Conclusions

This study shows that the full‐field strain maps on the cortical tibia bone surface vary in C57BL6/J mice with age and sex. This will make it possible for us to more precisely match strains to the ensuing changes in cellular osteogenic/antiresorptive adaptive activity between the various groups we analyzed. Understanding how mechano‐responsiveness is influenced by aging and sex will help understand changes in mechano‐responsiveness in aged bone.

## Disclosures

The authors have no conflict of interests.

## Author Contributions

**Alessandra Carriero:** Conceptualization; formal analysis; funding acquisition; investigation; methodology; project administration; resources; writing‐original draft preparation; writing–review & editing; visualization. **Behzad Javaheri:** Conceptualization; writing‐review & editing. **Neda Bassir Kazeruni:** Investigation; methodology; writing‐review & editing. **Andrew A. Pitsillides:** Conceptualization; funding acquisition; resources; writing‐review & editing. **Sandra J. Shefelbine:** Conceptualization; funding acquisition; project administration; resources; writing‐review & editing.

### Peer Review

The peer review history for this article is available at https://publons.com/publon/10.1002/jbm4.10467.
